# Redefining precision and efficiency in orthognathic surgery through virtual surgical planning and 3D printing: a narrative review

**DOI:** 10.1186/s40902-023-00409-2

**Published:** 2023-12-18

**Authors:** Yong-Chan Lee, Seong-Gon Kim

**Affiliations:** 1 Department of Oral and Maxillofacial Surgery, Bestian Dental Clinics, Seoul, 06218 Republic of Korea; 2 Department of Oral and Maxillofacial Surgery, College of Dentistry, Gangneung-Wonju National University, Gangneung, 28644 Republic of Korea

**Keywords:** Virtual surgical planning (VSP), 3D printing, Orthognathic surgery, Computer-aided design (CAD), Computer-aided manufacturing (CAM)

## Abstract

Orthognathic surgery, essential for addressing jaw and facial skeletal irregularities, has historically relied on traditional surgical planning (TSP) involving a series of time-consuming steps including two-dimensional radiographs. The advent of virtual surgical planning (VSP) and 3D printing technologies has revolutionized this field, bringing unprecedented precision and customization to surgical processes. VSP facilitates 3D visualization of the surgical site, allowing for real-time adjustments and improving preoperative stress for patients by reducing planning time. 3D printing dovetails with VSP, offering the creation of anatomical models and surgical guides, enhancing the predictability of surgical outcomes despite higher initial setup and material costs. The integration of VSP and 3D printing promises innovative and effective solutions in orthognathic surgery, surpassing the limitations of traditional methods. Patient-reported outcomes show a positive post-surgery impact on the quality of life, underlining the significant role of these technologies in enhancing self-esteem and reducing anxiety. Economic analyses depict a promising long-term fiscal advantage with these modern technologies, notwithstanding the higher initial costs. The review emphasizes the need for large-scale randomized controlled trials to address existing research gaps and calls for a deeper exploration into the long-term impacts and ethical considerations of these technologies. In conclusion, while standing on the cusp of a technological renaissance in orthognathic surgery, it is incumbent upon the medical fraternity to foster a collaborative approach, balancing innovation with scrutiny to enhance patient care. The narrative review encourages the leveraging of VSP and 3D printing technologies for more efficient and patient-centric orthognathic surgery, urging the community to navigate uncharted territories in pursuit of precision and efficiency in the surgical landscape.

## Background

Orthognathic surgery is a specialized branch of oral and maxillofacial surgery that focuses on correcting irregularities of the jaw and facial skeleton [1]. One of the most compelling reasons for the importance of orthognathic surgery lies in its ability to dramatically improve a patient’s quality of life through enhanced functionality [2]. Misaligned jaws can lead to a host of problems, including difficulties in chewing, speaking, and breathing [2, 3]. For instance, a patient with a severe underbite may struggle with efficiently chewing food, leading to digestive issues. Similarly, those with an open bite might find it challenging to articulate words clearly, affecting their social interactions and self-esteem. By realigning the jaws, orthognathic surgery can alleviate these functional impairments, allowing patients to eat, speak, and breathe more comfortably [2, 4].

Orthognathic surgery often involves an interdisciplinary approach, incorporating orthodontics, radiology, and even speech therapy to achieve the best possible outcomes [5]. This comprehensive care model underscores the surgery’s importance as it aims to address the patient’s overall well-being, rather than just isolated issues. The long-term health benefits of orthognathic surgery are also noteworthy. Correcting jaw misalignment can reduce wear and tear on the teeth, decrease the risk of temporomandibular joint (TMJ) disorders, and even mitigate sleep apnea in some cases [5, 6]. These long-term benefits contribute to the surgery’s overall importance, as they can prevent a cascade of health issues that might arise from untreated jaw irregularities.

Traditional surgical planning (TSP) for orthognathic surgery has been the standard approach for decades. This method involves a series of steps, including clinical examinations, two-dimensional radiographs, plaster model surgery, and the fabrication of surgical splints [7]. These steps are time-consuming but have been considered reliable for achieving satisfactory surgical outcomes [8]. Traditional methods primarily rely on two-dimensional imaging techniques like cephalometric radiographs [7, 8]. While these images provide valuable information, they lack the three-dimensional insight that is crucial for understanding complex anatomical relationships. This limitation can result in less accurate surgical planning and potentially compromise the surgical outcome [9]. Manual methods are susceptible to human error and can be inconsistent [9]. For example, the process of transferring the surgical plan to the operating room via surgical splints can introduce inaccuracies, affecting the result. Additionally, the skill level of the practitioner can significantly influence the quality of the planning and surgical outcome [8, 9].

The advent of computer-aided design (CAD) and computer-aided manufacturing (CAM) technologies has revolutionized various fields, including healthcare. Virtual surgical planning (VSP) and 3D printing are natural extensions of these advancements, offering unprecedented precision and customization in orthognathic surgery [10]. As demonstrated in recent studies, VSP significantly reduces the time required for surgical planning compared to traditional methods [7]. This time-saving aspect is not just beneficial for the surgical team but also minimizes the patient’s preoperative stress and waiting time.

The primary aim of this narrative review is to offer a concise yet comprehensive analysis of VSP and 3D printing in orthognathic surgery. The review seeks to evaluate the efficacy of these technologies in enhancing surgical accuracy, safety, and patient satisfaction. It will also scrutinize the time- and cost-efficiency of VSP and 3D printing compared to traditional methods. Further, the review will highlight recent technological advancements and their educational impact, especially for surgical trainees. Ethical considerations, such as patient consent and data security, will also be discussed. Lastly, the review aims to outline future research avenues and offer evidence-based recommendations for clinical practice. Overall, this review aspires to be an essential guide for healthcare professionals and policymakers interested in leveraging advanced technologies for improved surgical outcomes.

## Main text

### VSP

VSP is a cutting-edge technology that allows for the digital planning of surgical procedures, offering a 3D visualization of the surgical site [11]. In the context of orthognathic surgery, VSP is particularly relevant as it enables surgeons to simulate various surgical scenarios, assess potential outcomes, and make real-time adjustments [9]. This level of planning is crucial for complex procedures like orthognathic surgery, where millimeter-level precision can significantly impact both aesthetic and functional outcomes [12].

One of the most compelling advantages of VSP is its high level of accuracy and precision [12]. Several studies have corroborated this claim. For instance, a systematic review by Chen et al. [9] found that VSP was significantly more accurate in predicting postoperative outcomes compared to traditional methods. Another study by Alkhayer et al. [11] demonstrated that VSP could reduce the margin of error to less than 2 mm, thereby enhancing the surgical outcome.

Time is a critical factor in any surgical procedure, and VSP has been shown to offer significant time-saving benefits [13, 14]. Traditional surgical planning methods often involve labor-intensive processes like manual measurements and 2D imaging, which can be time-consuming. In contrast, VSP allows for quicker, more efficient planning. Studies have shown that VSP can reduce planning time by up to 30%, making it a more efficient alternative to traditional methods [13].

**Fig. 1:**
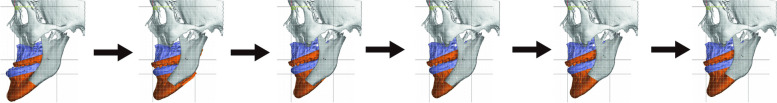
Simulation surgery utilizing the VSP system. A depiction of a surgical simulation conducted through the VSP system, illustrating the detailed planning process involved in orthognathic surgery

The implementation of VSP does require specific software and hardware. Software solutions often come with features like 3D visualization, real-time adjustments, and scenario simulations [15]. On the hardware side, a high-performance computer with a good graphics card is generally required for smooth operation [16]. Some setups also use haptic devices for a more interactive experience [17]. Various tools are employed in the VSP process, including 3D scanners for capturing high-resolution images of the surgical site, and 3D printers for creating physical models and surgical guides [15]. Software tools often include modules for soft tissue simulation, bone segmentation, and even predictive analytics for postoperative outcomes (Fig. 1).

There are several systems for VSP in the market (Table 1). When compared Dolphin Imaging and IPS Case Designer, Dolphin software exhibited superior performance in both single acquisition and long-run settings, showcasing more effective imaging [18]. However, Dolphin Imaging required 17 windows to complete planning, while IPS needed only 14 [18]. The difference in the number of windows suggests that IPS might offer greater ease of use. The comparison revealed that both Dolphin Imaging and IPS have their strengths and are largely comparable in many aspects, including acquisition times and the linearity of the programming path [18]. It suggests that the choice between the two could be based on individual preferences and specific needs, including considerations like operating system compatibility. Except for these systems, several other systems are available in the market (Table 1). There is currently no agreement on the most effective 3D prediction models [9]. The accuracy of soft tissue changes depicted in 3D prediction models may surpass that of TSP [9]. Therefore, it is important to delve into the strengths and limitations of various 3D virtual software systems available on the market. In the dynamic landscape of orthognathic surgery, VSP have been at the forefront in achieving precise surgical outcomes, including the accurate positioning of the condyle. A meticulous analysis of condyle positional changes can offer profound insights into the optimization of surgical strategies. VSP system facilitates the highest agreement between planned and actual outcomes for condylar positions, underlining the reduced scope of errors and the imperative of considering the propensity of surgical errors in different anatomical locations [19].

**Table 1:** Commercially available software for VSP

Software	Manufacturer	City, country
Dentofacial Planner Plus	Dentofacial Software	Toronto, ON, Canada
IPS CaseDesigner	KLS Martin Group	Jacksonville, FL, USA
Quick Ceph	Quick Ceph Systems	San Diego, CA, USA
Dolphin Imaging	Dolphin Imaging Software	Canoga Park, CA, USA
BOS system	Spenser Biomedical Technology	Seoul, Korea

By offering high levels of accuracy, time efficiency, and adaptability, VSP stands as a revolutionary tool in the realm of orthognathic surgery [9, 12]. Its growing adoption is a testament to its effectiveness and the tangible benefits it offers to both surgeons and patients alike [10, 20].

### 3D printing

**Fig. 2:**
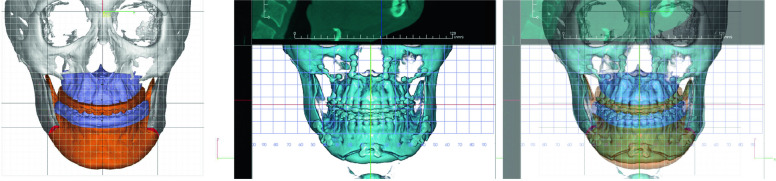
Accuracy evaluation through comparative analysis. A comparative analysis showcasing the simulated surgery outcome (left) alongside the actual surgery result (middle). The overlay of these images (right) facilitates the assessment of the VSP system’s accuracy

3D printing, also known as additive manufacturing, is a technology that allows for the creation of three-dimensional objects from digital models [21]. In the realm of orthognathic surgery, 3D printing has found a variety of applications, including the fabrication of surgical guides, anatomical models for preoperative planning, and even custom implants [10]. These applications enhance the precision and predictability of surgical outcomes, thereby elevating the standard of care (Fig. 2).

Several types of 3D printing technologies are commonly used in medical settings:

1. Fused deposition modeling (FDM): This is one of the most accessible and cost-effective methods, suitable for creating fewer complex models [22].
2. Stereolithography (SLA): Known for its high resolution and accuracy, SLA is often used for intricate structures like vascular networks [23].
3. Selective laser sintering (SLS): This method is used for more robust and durable models, as it fuses powder layers via a laser [24].

Each of these technologies has its own set of advantages and limitations, making them suitable for specific applications in orthognathic surgery [10].

The choice of material is crucial in 3D printing for medical applications. Commonly used materials include the following:

1. Polylactic acid (PLA): Biodegradable and safe for human contact, often used for temporary implants or surgical guides [25].
2. Resins: Used in SLA printing, these offer high detail but are generally less durable than other materials [26].
3. Nylon: Known for its strength and durability, it is often used in SLS printing for more robust surgical tools or models [27].

The advantages of 3D printing can be enlisted as follows.

**Fig. 3:**
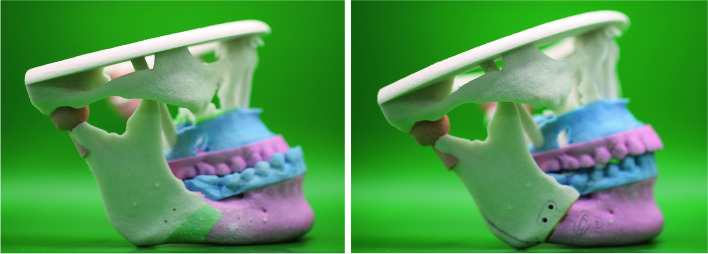
Portraying a patient-specific rapid prototyping (RP) model, showcasing the preoperative state on the left and the postoperative state on the right. These models are created through the utilization of RP technology, highlighting the practical applications of 3D printing in surgical preparations. The preoperative state, depicted on the left side of the figure, represents the initial condition of the patient’s anatomy. Through the RP model, surgeons can accurately visualize the patient’s anatomy prior to the surgical procedure. On the right side of the figure, the postoperative state is illustrated. The RP model aids in depicting the desired outcome of the surgical intervention. Surgeons can use the model to simulate the surgical procedure and assess the feasibility of their proposed surgical plan. By comparing the preoperative and postoperative states, surgeons can evaluate the effectiveness of their surgical technique and make any necessary adjustments prior to the actual surgery. This enables them to optimize surgical outcomes and minimize potential complications

1. Precision: 3D printing allows for highly accurate models, which can be crucial for complex surgeries [14].
2. Customization: Surgeons can create patient-specific models, surgical guides, and prebend plates, enhancing the individualized approach to treatment (Fig. 3) [15].
3. Time-efficiency: Once the digital model is ready, multiple copies can be printed with minimal additional time investment [11].

However, 3D printing has some limitations as follows [9, 11].

1. Cost: Initial setup and material costs can be high, although these may be offset by time and resource savings in the long run.
2. Technical expertise: Operating 3D printers and designing models require specialized skills.
3. Material limitations: Not all materials are suitable for every medical application, and some may require post-processing or sterilization.

In summary, 3D printing offers a range of benefits that make it a valuable tool in orthognathic surgery, despite some limitations. Its ability to produce precise, customized surgical aids represents a significant advancement over traditional methods.

### Comparative analysis

In recent years, numerous studies have been conducted to compare VSP with traditional methods in orthognathic surgery [9–11]. The consensus leans towards VSP offering a more precise and efficient approach [12]. It has been noted that VSP allows for a meticulous preoperative plan, reducing the time spent during surgery and potentially leading to better outcomes [11]. Moreover, it facilitates a collaborative environment where multidisciplinary teams can work together seamlessly, enhancing the planning process significantly [7]. However, it is essential to note that the learning curve associated with the adoption of new technology can initially prolong the planning phase, especially for individuals with limited experience in digital technologies.

In a comparative analysis, it has been found that the VSP technique exhibits greater precision in predicting soft tissue changes compared to the TSP technique. This is evidenced by the fact that the vertical and horizontal mean differences in soft tissue changes observed with the VSP technique are below 1.50 mm, while those with the TSP technique exceed 2.00 mm [28–30]. Moreover, the VSP technique utilizes various mathematical prediction models within its virtual planning software [31]. However, there is currently no consensus regarding the optimal 3D prediction models to be used in this technique.

It can be suggested, though, that incorporating 3D models in the prediction process may yield closer approximations to the actual soft tissue changes. As a result, the VSP technique holds promise as a more precise approach to predicting soft tissue changes in orthognathic surgery, thus offering greater clinical value [9]. However, it is worth noting that both VSP and TSP techniques exhibit similar surgical accuracy for hard tissue in the sagittal plane [32]. To further enhance the reliability and efficacy of the VSP technique, further empirical studies are required to validate and refine the prediction models that are currently utilized.

3D printing has revolutionized the field of orthognathic surgery, offering a range of benefits over traditional methods. In the conventional approach, surgeons typically rely on the ANS, PNS, and point A as reference points during surgery [33]. These points, however, can be removed during the surgical procedure, posing a risk of losing vital reference markers, which can potentially lead to inaccuracies in the surgical outcome [34]. The 3D printing-assisted approach mitigates the issue of losing reference points by utilizing the nasal notch of the maxilla bone as a reference. This strategy ensures a more stable and reliable reference point, aiming to reduce the potential errors that can occur due to the removal of traditional reference points, thus promising a more accurate surgical procedure [33].

The conventional method often employs intermaxillary wafers to position the maxilla based on the mandible, a technique that can introduce postoperative variations and has been noted to frequently lead to errors [35]. The use of face bow transfers to record the relationship between the maxilla and the hinge axis of mandible rotation has been shown to have inherent inaccuracies [36]. Studies cited in the discussion indicate a significant potential for error in the angle of the occlusal plane during the transfer process, which can adversely affect the surgical outcomes [33]. The modern approach leverages 3D virtual simulations and 2D cephalometric analyses to facilitate accurate preoperative planning. This technique promises to reduce the errors commonly seen in the conventional methods that use articulators for planning [33]. The 3D printing technology enables the creation of patient-customized osteotomy guides and plates, enhancing the precision in replicating the virtual surgery plans during the actual surgical procedure [15]. This approach seeks to overcome the limitations of conventional methods, offering a pathway to more accurate and reliable surgical outcomes [33]. However, the high initial costs and the need for specialized training are often cited as limitations [11]. Despite these, the consensus is that the benefits outweigh the drawbacks, with 3D printing being an asset in modern surgical planning.

When VSP and 3D printing are used in tandem, they offer synergistic benefits that can significantly enhance the outcomes in orthognathic surgery [9]. The combination allows for a seamless transition from virtual planning to the creation of physical models and surgical guides, ensuring a high level of accuracy and predictability [12]. This synergy facilitates a more streamlined and efficient workflow, reducing the chances of errors and the time required for surgery [11]. Moreover, it fosters a patient-centric approach, where customized solutions can be developed to address individual needs, thereby potentially improving patient satisfaction and outcomes [9]. The integration of these advanced technologies represents a paradigm shift in orthognathic surgery, paving the way for more innovative and effective solutions in the future.

### Patient-reported outcomes

In the evolving landscape of orthognathic surgery, an increasingly pivotal role is being played by patient-reported outcomes in determining the success and efficacy of both traditional and advanced techniques, such as VSP and 3D printing. These outcomes predominantly focus on post-surgery quality of life, satisfaction with surgical results, and other patient-centric metrics that holistically depict the patient’s journey through the surgical process [37, 38].

An array of studies has focused on the trajectories of quality of life post orthognathic surgery, leveraging both general health and condition-specific approaches to gauge the changes in patient experiences. Choi et al. [39] noted significant alterations in both physical and mental health scores post-treatment, pointing to a tangible impact on the quality of life. A distinct pattern emerges from various research where a considerable number of patients reported improved quality of life in both functional and psychological domains after undergoing the surgery [37, 40]. It is notable that the improvements were more pronounced in older patients and those who underwent double-jaw surgery, especially in the context of class III malocclusion [40].

Delving deeper into patient satisfaction, a consensus emerges across studies that most individuals report a high level of satisfaction following orthognathic surgeries. Studies have cited enhancements in areas such as self-esteem, self-confidence, and satisfaction with facial appearance, coupled with a reduction in anxiety and social functioning issues [41, 42]. However, it is crucial to note a minority who remain dissatisfied, pointing to a spectrum of responses possibly influenced by individual personality traits, background, and relational dynamics. This underlines the necessity for a nuanced approach in patient consultations to foster realistic expectations and understanding [41].

As technology steadily permeates the surgical sphere, its impact on patient outcomes warrants scrutiny. Hanafy et al. [43] embarked on a comparative study between CAD/CAM bone splints and traditional occlusal wafers, finding that while both groups exhibited improved quality of life post-surgery, the technological intervention did not significantly outperform the traditional approach. This finding reverberates the necessity to balance rapid technological advancements with grounded expectations and to continually evaluate the real-world impacts of these advancements on patient outcomes.

Moving forward, there is a concerted call in the scientific community for research designs bearing higher levels of evidence, encompassing larger and diverse patient groups, and extending follow-up durations to forge a more robust understanding of the long-term impacts of orthognathic surgeries on quality of life [37, 38]. Additionally, there is a growing recognition of the importance of psychological support during treatment, and understanding the processes of adjustment to facial changes post-surgery, indicating a trajectory towards a more holistic approach to patient care in orthognathic surgery [42].

In conclusion, patient-reported outcomes serve as a crucial lens to evaluate the evolving landscape of orthognathic surgery, offering rich insights into the lived experiences of patients. While advancements in technology herald a new era in surgical interventions, the core of patient satisfaction and improved quality of life remains a multifaceted construct, influenced by a gamut of factors including psychological preparedness, realistic expectations, and individual health trajectories. Thus, a nuanced understanding of these outcomes, drawn from a rich tapestry of patient narratives and statistical evidence, stands central to steering the future directions in orthognathic surgical interventions.

### Cost analysis

In the continuously evolving field of orthognathic surgery, evaluating the economic ramifications of implementing modern technologies such as VSP and 3D printing compared to conventional approaches is crucial. This section explores a meticulous cost analysis, investigating diverse aspects including initial setup costs, operational expenses, and the prospective for long-term fiscal advantages drawn from several recent studies.

Bengtsson et al. [44] revealed no significant difference in the total time spent in both techniques; however, the 2D method showcased a substantial financial advantage, necessitating lower radiation doses. Yet, the 3D technique incurred an escalated economic cost per health-related quality of life point gained, highlighting a trade-off between financial costs and radiation dose. Schneider et al. [45] further endorsed the financial implications of adopting modern technologies in their prospective randomized trial. The study delineated that while VSP exhibited a superior accuracy in treatment planning and a reduction in operation duration, it also augmented the total planning costs significantly [45]. Despite the heightened costs, the study hinted at the potential of these virtual methodologies eventually replacing traditional orthognathic surgery as they become cost-effective.

Resnick et al. [46] carried out a retrospective cohort study, contrasting the costs between VSP coupled with 3D printing of splints and standard planning involving 2D cephalometric evaluations and manual splint fabrication. The findings were in favor of VSP, denoting a significant reduction in both time and costs across various case types analyzed, contrary to the common notion of escalated costs with advanced technologies [46]. Park et al. [47] also ventured into a retrospective study to compare the time and costs between TSP and VSP in Korea. The research illustrated a notable diminution in the time invested in VSP compared to TSP, particularly more pronounced in surgeries involving Le Fort I osteotomy combined with bilateral sagittal split osteotomy [47]. However, the study did not witness a statistically significant reduction in costs, bringing to light the time-saving attribute of VSP without a corresponding financial benefit.

Synthesizing the insights from these studies, it is evident that the financial landscape of orthognathic surgery is witnessing a paradigm shift with the introduction of modern 3D technologies. While these technologies offer substantial time savings and enhanced accuracy, they do incur a higher initial financial outlay compared to traditional 2D methods. However, when viewed from a broader perspective, including the potential for improved health-related quality of life and reduced operation times, the economic argument in favor of 3D technologies gains substantial ground.

Looking forward, it is imperative to undertake a more nuanced analysis, encompassing a wider array of case types and considering long-term financial implications to forge a comprehensive understanding of the cost dynamics. As the technology matures and becomes more prevalent, it is plausible that economies of scale may come into play, further enhancing the cost-effectiveness of these advanced technologies in orthognathic surgery. The trajectory indicates a promising future, balancing economic efficiency with technological advancement in shaping the future of orthognathic surgical interventions.

### Limitations and future directions

In the process of reviewing the existing literature on VSP and 3D printing in orthognathic surgery, several limitations have been noted. Many studies have small sample sizes, which can potentially introduce bias and limit the generalizability of the findings [9, 11]. Additionally, there is a notable scarcity of randomized controlled trials (RCTs), which are the gold standard for evaluating the effectiveness of interventions. Most of the studies are retrospective in nature, which can introduce selection bias and affect the quality of the results. Furthermore, there is a variation in the methodologies employed across different studies, making it challenging to compare results directly. Some studies focus exclusively on specific aspects such as time efficiency or accuracy, without offering a comprehensive view that encompasses all relevant factors including patient satisfaction and economic implications.

Current research presents a fragmented picture with significant gaps that need to be addressed to provide a more rounded understanding of the field. One of the glaring gaps is the limited exploration of the ethical dimensions associated with the adoption of advanced technologies, including issues pertaining to patient consent and data security. Moreover, there is a lack of studies investigating the long-term outcomes of surgeries planned using VSP and 3D printing technologies, including impacts on patients’ quality of life and satisfaction over extended periods. The economic analysis of the adoption of these technologies is also somewhat underexplored, with a need for more detailed studies examining the cost–benefit dynamics over the long term.

Looking forward, it is imperative to address the identified gaps and limitations in the existing body of research to foster a deeper understanding of the potential and challenges associated with VSP and 3D printing in orthognathic surgery. Future research should prioritize conducting RCTs with larger sample sizes to yield more robust and generalizable findings. There is a pressing need to delve deeper into the ethical considerations and to develop frameworks that ensure the responsible adoption of these technologies. Moreover, studies should explore the potential of these technologies in medical education, particularly in enhancing the training experience for surgical trainees and junior surgeons. Furthermore, research should focus on the continual advancements in VSP and 3D printing technologies, including exploring new materials and techniques that can further enhance the accuracy and efficiency of surgical planning and execution.

## Conclusion

The advent of VSP and 3D printing technologies marks a transformative era in orthognathic surgery, offering remarkable advancements in surgical precision, efficiency, and patient-centered outcomes. This narrative review underscores the considerable potential of these technologies to improve surgical accuracy, reduce operative times, and enhance patient satisfaction, thereby setting a new standard in orthognathic surgical care.

Despite these significant advancements, challenges and limitations remain, particularly in the realms of cost, technological adoption, and the need for comprehensive research. The dearth of large-scale randomized controlled trials and fragmented nature of current studies highlight the necessity for more rigorous research in this evolving field. Ethical and economic considerations of integrating these technologies into healthcare require further exploration to ensure their responsible and sustainable use. As the medical community navigates this technological renaissance, a balanced approach is essential, embracing innovation while critically evaluating potential drawbacks. Collaboration across disciplines will be crucial in optimizing the use of VSP and 3D printing for the betterment of patient care.

In conclusion, VSP and 3D printing are pivotal in the evolution of orthognathic surgery. Future research directions should focus on addressing current gaps, further refining these technologies, and solidifying their role in advancing surgical practice. The promise of these technologies lies in their ability to enhance surgical precision and patient outcomes, heralding a new chapter in orthognathic surgery.

## Data Availability

Data sharing is not applicable to this article since no dataset was generated or analyzed during the current study.

## Abbreviations

CAD/CAM: Computer-aided design and manufacturing
FDM: Fused deposition modeling
PLA: Polylactic acid
RCTs: Randomized controlled trials
SLA: Stereolithography
SLS: Selective laser sintering
TMJ: Temporomandibular joint
TSP: Traditional surgical planning
VSP: Virtual surgical planning
